# A Strategy for Functional Interpretation of Metabolomic Time Series Data in Context of Metabolic Network Information

**DOI:** 10.3389/fmolb.2016.00006

**Published:** 2016-03-07

**Authors:** Thomas Nägele, Lisa Fürtauer, Matthias Nagler, Jakob Weiszmann, Wolfram Weckwerth

**Affiliations:** ^1^Department of Ecogenomics and Systems Biology, University of ViennaVienna, Austria; ^2^Vienna Metabolomics Center, University of ViennaVienna, Austria

**Keywords:** metabolic network, data integration, metabolomics, time series analysis, systems biology, network dynamics

## Abstract

The functional connection of experimental metabolic time series data with biochemical network information is an important, yet complex, issue in systems biology. Frequently, experimental analysis of diurnal, circadian, or developmental dynamics of metabolism results in a comprehensive and multidimensional data matrix comprising information about metabolite concentrations, protein levels, and/or enzyme activities. While, irrespective of the type of organism, the experimental high-throughput analysis of the transcriptome, proteome, and metabolome has become a common part of many systems biological studies, functional data integration in a biochemical and physiological context is still challenging. Here, an approach is presented which addresses the functional connection of experimental time series data with biochemical network information which can be inferred, for example, from a metabolic network reconstruction. Based on a time-continuous and variance-weighted regression analysis of experimental data, metabolic functions, i.e., first-order derivatives of metabolite concentrations, were related to time-dependent changes in other biochemically relevant metabolic functions, i.e., second-order derivatives of metabolite concentrations. This finally revealed time points of perturbed dependencies in metabolic functions indicating a modified biochemical interaction. The approach was validated using previously published experimental data on a diurnal time course of metabolite levels, enzyme activities, and metabolic flux simulations. To support and ease the presented approach of functional time series analysis, a graphical user interface including a test data set and a manual is provided which can be run within the numerical software environment Matlab®.

## Introduction

The functional interpretation of experimental data in context of biochemical network information represents one of the central challenges in current biological research. While genome sequencing projects have enabled the reconstruction of genome-scale metabolic networks, their high dimensionality precludes a direct and intuitive application to interpret experimental data. Hence, although genome sequence information and metabolic networks have become available for numerous organisms, tissues, or cell types (Herrgard et al., 2008; Chang et al., 2011; De Oliveira Dal'Molin and Nielsen, 2013; Thiele et al., 2013), functional metabolic data interpretation still represents a major obstacle in systems biology. Various mathematical and computational strategies from the fields of multivariate statistics, ordinary, and partial differential equations (ODEs/PDEs), optimization or statistical time series analysis have been developed and applied to reveal a biologically meaningful interpretation of comprehensive and multidimensional experimental data sets. For example, a computational model of starch metabolism in plants enabled the analysis of starch kinetics through diurnal metabolic and circadian sensors (Pokhilko et al., 2014). The authors developed a model of 28 ODEs which were numerically simulated in order to analyze diurnal kinetics of carbon metabolism *in silico*. In another study, the response of *Escherichia coli* to varying oxygen concentrations was analyzed applying a mathematical model of the central metabolism (Ederer et al., 2014). Here, the authors derived a prediction about the impact of product formation on biomass concentration using steady state simulations at varying environmental conditions.

Both examples for mathematical modeling differ in organism and application. Besides, the dynamic approach can be distinguished from the steady state approach. However, in both approaches, dynamics of metabolic systems can be described by sets of ODEs. If sufficient kinetic information is available, such ODEs can be numerically integrated revealing simulated metabolic concentrations depending on time, enzyme parameters, thermodynamic constraints, etc. Yet, statistically robust experimental enzyme kinetic information often limits the applicability of such modeling approaches. Particularly, the resolution of enzyme activities, substrate affinities, or inhibitory constants is very laborious and only possible if well-established experimental assays and sufficient biochemical knowledge are available. Additionally, uncertainties about model structures and reaction kinetics complicate the interpretation of a numerically simulated output (Schaber et al., 2009). Such limitations have been addressed by different theoretical approaches, for example by structural kinetic modeling, SKM (Steuer et al., 2006). In the SKM approach, local linear models are applied to explore and statistically analyze a given parameter space without the need for explicit information about functional forms of enzyme kinetics and rate equations. Finally, a Jacobian matrix is derived which characterizes the dynamic capabilities of a metabolic system at a certain steady state. In previous publications, we have developed a procedure to determine Jacobian matrices directly from experimental metabolomics data (Nägele, 2014; Nägele et al., 2014). Based on experimental metabolic (co)variance information a metabolic regulator was identified indicating a strategy how plant metabolism is reprogrammed during exposure to energy limiting conditions. In a different context, other studies have also shown that it is possible to infer regulatory information about metabolic steady states from experimental data with such approaches (see e.g., Steuer et al., 2003; Sun and Weckwerth, 2012; Kügler and Yang, 2014).

Beyond these approaches of dynamic and steady state modeling, time series analysis and related regression models offer another mathematical strategy to reveal information about molecular system dynamics (Schelter, 2006). For example, Dutta and co-workers developed an algorithm for identification of differentially expressed genes in a time series experiment (Dutta et al., 2007), which they also applied to integrate transcriptome and metabolome data (Dutta et al., 2009). In another study, statistical modeling and regression analysis revealed a nitrogen-dependent modulation of root system architecture in the genetic model plant *Arabidopsis thaliana* (Araya et al., 2015). While these exemplarily mentioned studies present only a very small fraction of possible statistical applications, it already becomes evident that these are promising and necessary mathematical approaches to reveal biologically meaningful information from comprehensive experimental data sets being preliminary for hypothesis generation and experimental validation. However, a common problem of regression and correlation approaches in a biochemical context is a missing functional linkage of the results to causal biochemical interrelations, i.e., enzymatically driven reactions. To overcome this limitation and to facilitate the biochemical interpretation of the statistical results, the present study derives a theoretical connection between mathematical approaches of ODE-based dynamic modeling and statistical time series analysis. Based on the stoichiometric matrix information of a metabolic network, ratios of time-dependent derivative functions were built providing an estimate for the strength and probability of a metabolic interaction during the time course. The suggested strategy was tested using previously published experimental data sets on diurnal and stress-induced dynamics of metabolite concentrations and related enzyme kinetic information. Finally, a graphical user interface for Matlab is provided which intends to facilitate the application of the presented strategy.

## Results

### Deriving metabolic functions by inverse variance-weighted regression analysis

Time-dependent dynamics of metabolite concentrations in a biochemical network can be described by a set of ODEs:

(1) $$\frac{d}{dt}M\left(t\right)=\mathrm{Nv}\left(M,p,t\right)=f(M,p,t)$$

Here, ***M*** represents an n-dimensional vector of mean metabolite concentrations (c_*n*_), ***N*** is the *n* × *k* stoichiometric matrix and ***v*** describes the *k*-dimensional vector of reaction rates which depend on metabolite concentrations ***M***, enzyme kinetic parameters ***p*** and time *t*. The right side of the ODE system can be summarized by metabolic functions, *f(****M,p****,t)*. Hence, these metabolic functions define the time-dependent changes in metabolite concentration as a sum of all biochemical reactions either consuming or producing a metabolite. A metabolic steady state is described by ODEs which equal zero:

(2) $$\frac{d}{dt}M\left(t\right)=0$$

Linearization enables the investigation of the system behavior close to a steady state. The linearization process results in the so-called Jacobian matrix ***J*** which characterizes the dynamic properties of the system at a steady state:

(3) $$J=\left(\begin{array}{c} \frac{\partial f_{1}}{\partial c_{1}}\cdots \frac{\partial f_{1}}{\partial c_{n}}\\ \vdots \ddots \vdots \\ \frac{\partial f_{n}}{\partial c_{1}}\cdots \frac{\partial f_{n}}{\partial c_{n}} \end{array}\right)$$

Hence, in a biochemical context, the Jacobian matrix ***J*** describes the behavior of metabolic functions *f*_*i*_ (for *i* = 1,…,n) of a metabolic network with regard to small changes of variables *c*_*i*_ (for *i* = 1,…,n), i.e., metabolite concentrations at the considered steady state. The information if a metabolic function *f*_*i*_ biochemically depends on the concentration of a metabolite c_*i*_ is provided by the stoichiometric matrix ***N*** of a metabolic network (see Equation 1).

To derive, i.e., predict, time continuous information from time discrete experimental observations, interpolation methods can be applied. To prevent unrealistic oscillations of high-degree polynomial interpolation, intervals of approximation can be partitioned in subintervals which can be approximated, for example, by cubic polynomials which form a cubic spline *S*_*c_i_*_(*t*) (see e.g., Bronstein et al., 2008):

(4) $$S_{c_{i}}\left(t\right)=\alpha_{ij}+\beta_{ij}\left(t-t_{j}\right)+\gamma_{ij}{\left(t-t_{j}\right)}^{2}+\delta_{ij}{\left(t-t_{j}\right)}^{3}$$

Here, it is *t* ∈ [*t*_*j*_, *t*_*j*+1_] with (*j* = 1, 2,…, z−1), where z represents the number of interpolation nodes (*t*_*j*_, *g*_*ij*_*)*, and it is *S*_*c_i_*_(*t_j_*) = *g*_*ij*_. Interpolation coefficients are represented by α, β, γ, and δ. Due to the occurrence of experimental errors, the requirement of *S_c_i__*(*t_j_*) = *g*_*ij*_ is not fulfilled which prevents the suitability of such a type of interpolation. Instead, a smoothing element can be introduced accounting for those experimental errors:

(5) $$min\left(\sum_{j=1}^{z}w_{ij}{\left[g_{ij}-S_{c_{i}}(t_{j})\right]}^{2}+\lambda \int_{t=t_{1}}^{t_{z}}{\left[S_{c_{i}}^{\prime \prime }(t)\right]}^{2}dt\right)$$

Here, *w*_*ij*_ represents a weighting factor, $S_{c_{i}}^{{}^{\prime \prime }}\left(t\right)$ is the second derivative of *S_c_i__*(*t*), and λ (with λ ≥ 0) represents a smoothing factor. For λ = 0, one obtains the cubic spline interpolation, while the degree of smoothing increases with the value of λ. To connect the smoothing spline generation to experimentally observed errors we defined the weighting factor *w*_*ij*_ to equal the inverse variance information, i.e., the inverse squared standard deviation:

(6) $$w_{ij}=\sigma_{ij}{}^{-2}={\left(\frac{1}{r-1}\sum_{k=1}^{r}{\left(c_{ij,k}-{\bar{c}}_{ij}\right)}^{2}\right)}^{-1}$$

Here, *r* represents the number of experimental and independent replicates.

Merging Equations (1), (5) and (6) and replacing g_*ij*_ by the mean concentration of metabolite *i* at time point *j*, *c*_*ij*_, reveals a description of metabolic functions by inverse variance-weighted regression analysis:

(7) $$\begin{array}{l} \frac{d}{dt}M_{i}\left(t\right)=f_{i}\left(M,p,t\right)\\ \;=\frac{d}{dt}[min({\sum_{j=1}^{z}[\left(\frac{1}{r-1}\sum_{k=1}^{r}{\left(c_{ij,k}-{\bar{c}}_{ij}\right)}^{2}\right)}^{-1}\\ \;{\left[{\bar{c}}_{ij}-S_{c_{i}}(t_{j})\right]}^{2}]+\lambda \int_{t=t_{1}}^{t_{z}}{\left[S_{c_{i}}\prime \prime (t)\right]}^{2}dt)] \end{array}$$

**Figure 1:**
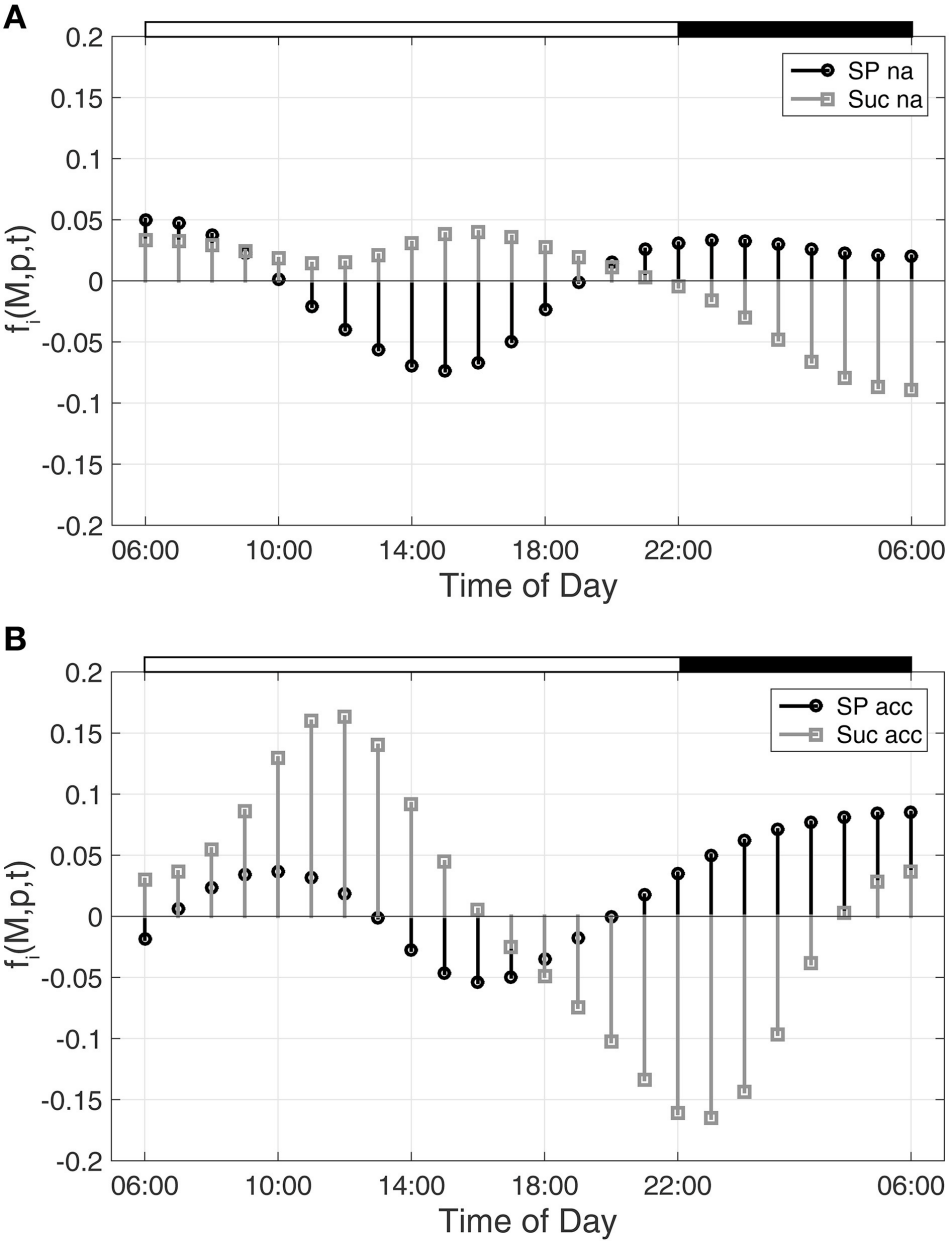
**Metabolic functions derived from inverse variance-weighted regression analysis for (A) non-cold acclimated and (B) cold acclimated plants**. Metabolic functions *f*_*i*_*(****M,p****,t)* were derived for sugar phosphates (SP) and sucrose (Suc) as described in the text (see Equation 7). Experimental data were derived from a previous study comprising metabolite levels of non-cold acclimated (na) and cold acclimated (acc) leaf material of *Arabidopsis thaliana*, accession Col-0 (Nägele et al., 2012). White and black bars on the top indicate light and dark phase of a diurnal cycle.

Hence, building the first derivative of the smoothed interpolation of experimental time-course data reveals information about the connected metabolic function. In the present study, this approach was applied to evaluate a diurnal time course of previously published metabolite concentrations (Nägele et al., 2012) belonging to the central carbohydrate metabolism in leaves of the genetic model plant *A. thaliana.* Diurnal dynamics of metabolic functions are shown exemplarily (Figure 1) for the metabolite pools of sucrose (Suc) and sugar phosphates (SP) in a control experiment (non-cold acclimated, na) and after exposure to low temperature (acc).

To characterize time-dependent changes in metabolic functions, the second time-dependent derivative was built from the approximated diurnal time course of metabolite concentrations:

(8) $$\begin{array}{l} \frac{d^{2}}{dt^{2}}M_{i}(t)=\frac{d}{dt}f_{i}(M,p,t)\\ \;=\frac{d^{2}}{dt^{2}}[min(\sum_{j=1}^{z}[{\left(\frac{1}{r-1}\sum_{k=1}^{r}{\left(c_{ij,k}-{\bar{c}}_{ij}\right)}^{2}\right)}^{-1}\\ \;\;\;{\left[{\bar{c}}_{ij}-S_{c_{i}}(t_{j})\right]}^{2}]+\lambda \int_{t=t_{1}}^{t_{z}}{\left[S_{c_{i}}^{\prime \prime }(t)\right]}^{2}dt)] \end{array}$$

**Figure 2:**
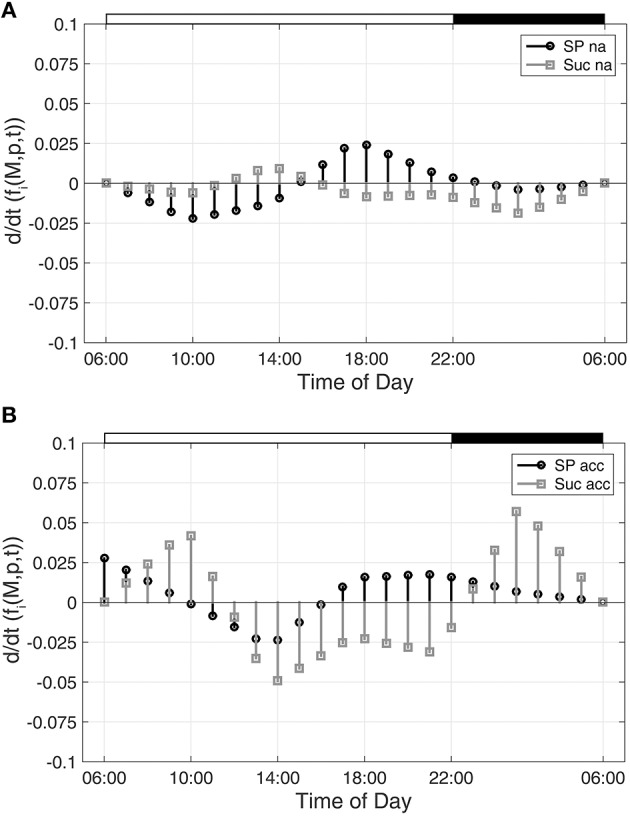
**Time-dependent dynamics of metabolic functions derived from inverse variance-weighted regression analysis for (A) non-cold acclimated and (B) cold acclimated plants**. Function dynamics were derived for sugar phosphates (SP) and sucrose (Suc) as described in the text (see Equation 8). Experimental data were derived from a previous study comprising metabolite levels of non-cold acclimated (na) and cold acclimated (acc) leaf material of *A. thaliana*, accession Col-0 (Nägele et al., 2012). White and black bars on the top indicate light and dark phase of a diurnal cycle.

As described for Figure 1, diurnal dynamics of those time-dependent changes of metabolic functions are also shown exemplarily for Suc and SP (Figure 2).

### Connecting metabolic functions based on biochemical network information

**Figure 3:**
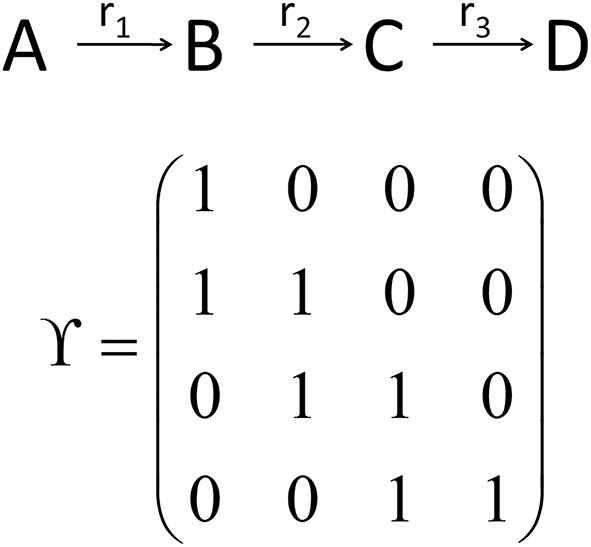
**Schematic reaction chain and the derived interaction matrix Y**. Rows (metabolic functions) and columns (metabolites) of **Y** describe biochemical interactions of metabolites A (first row/column), B (second row/column), C (third row/column), and D (fourth row/column). Entries, i.e., 0 and 1, indicate if two metabolites interact (entry 1) or not (entry 0).

While the metabolic time-course information derived before characterizes time-dependent rates of changes in each considered metabolite concentration separately (see Equations 7 and 8), information of biochemical interdependencies, i.e., the information about a substrate - product relationship between two or more metabolites, is only contained implicitly. To explicitly analyze and visualize these biochemical interdependencies with regard to the time-dependent rates of concentrations changes, a metabolic *n* × *n* interaction matrix, ***Y***, was derived where n represents the number of metabolites comprised by the model. In ***Y***, each entry indicates whether two metabolites are biochemically connected (entry: 1) or not (entry: 0). The interaction is characterized analogous to entries of the Jacobian matrix (Equation 3): if the metabolic function of metabolite A is biochemically connected to changes in concentrations of metabolite B, the corresponding entry in ***Y*** is 1. Information about metabolic functions is given row-wise, while biochemically connected metabolites are indicated column-wise for each function. In a simple example, containing three reactions (r_1_–r_3_) and four metabolites (A–D), the construction and content of ***Y*** is exemplified (Figure 3). The diagonal entries indicate the biochemical dependencies of metabolic functions on substrate concentrations. For example, ***Y***_*11*_ = 1 indicates that metabolic function f(A,t) depends on the concentration of A(t). The non-diagonal entries describe interdependencies between different metabolite pools. For example, ***Y***_*21*_ = 1 indicates that metabolic function f(B,t) depends on the concentration of A(t).

Based on a previously published metabolic model (Nägele et al., 2012), an interaction matrix ***Y*** was derived for the central carbohydrate metabolism in leaves of *A. thaliana*. The metabolic functions (Equation 7) and their time-dependent derivatives (Equation 8) were related to each other according to the entries of ***Y***. This finally resulted in functions ω(*a* → *b, t*) indicating changes in metabolic functions of *b* in context of concentration changes of *a* which might represent substrates, inhibitors or activators:

(9) $$\omega \left(a\to b,t\right)=\frac{\frac{d}{dt}f_{b}(M,p,t)}{f_{a}(M,p,t)},\;D=\left\{\mathbb{R}\;\backslash f_{a}\left(M,p,t\right)=0\right\}$$

With regard to the analyzed time-course of sugar phosphate (SP) and sucrose (Suc) concentrations (see Figures 1, 2), ω(SP → Suc, t) revealed information about the reaction of sucrose biosynthesis, catalyzed by the enzyme sucrose phosphate synthase (SPS):

(10) $$SugarPhosphates\left(SP\right)\;\overset{SPS}{\to }\;Sucrose$$

In detail, ω(SP→Suc, t) described changes in the metabolic function of sucrose in context of concentration changes of its biochemical substrate sugar phosphates:

(11) $$\omega \left(SP\to Suc,t\right)=\frac{\frac{d}{dt}f_{Suc}(M,p,t)}{f_{SP}(M,p,t)}$$

**Figure 4:**
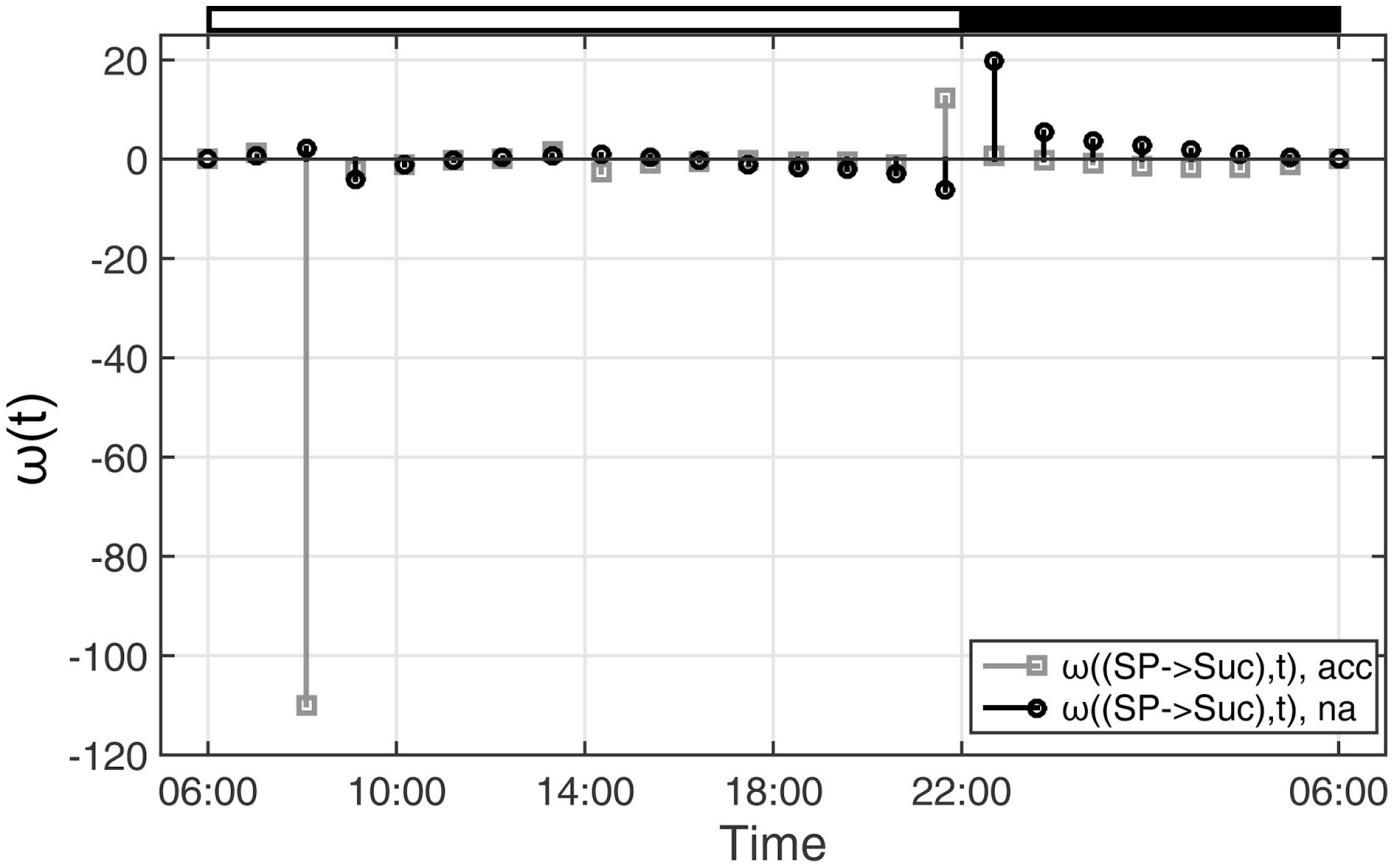
**Functions ω(t) indicating changes in metabolic functions in context of concentration changes of biochemical interaction partners**. ω(t) was calculated as described in the main text (see Equation 9). Results of ω(t) are shown for the reaction of sucrose biosynthesis for non-cold acclimated (na) and cold acclimated (acc) leaf material of *A. thaliana*, accession Col-0 (Nägele et al., 2012). White and black bars on the top indicate light and dark phase of a diurnal cycle.

Comparing ω(SP→Suc, t) for na and acc plants revealed a noticeable difference between both conditions within the first 4 h of the day (Figure 4). Interestingly, in the same time period, simulations of sucrose biosynthesis, based on a system of ordinary differential equations (ODEs), revealed a similar picture in which rates of sucrose biosynthesis were decreased only in acc plants (Nägele et al., 2012).

### Characterization of ω(t)

ω(*t*) represents a ratio of metabolic functions and related derivatives. Hence, the unit of ω(t) is derived from the flux unit of a metabolic function, [mM s^−1^]:

(12) $$\left[\omega \left(a\to b,t\right)\right]=\frac{\left[\frac{d}{dt}f_{b}\left(M,p,t\right)\right]}{\left[f_{a}\left(M,p,t\right)\right]}=\frac{\left[mM\;s^{-2}\right]}{\left[mM\;s^{-1}\right]}=\left[\frac{1}{s}\right]$$

Here, concentrations are given in mM (mmol l^−1^) and the time unit is seconds (s).

This results in the unit of a rate or frequency. Hence, |ω(*a*→*b,t*)| was interpreted as oscillations of a metabolic function per time-period with reference to a biochemical effector.

In the case of |ω(a → b, t)| → ∞ for *t* → τ, the influence of the biochemical effector on a metabolic function was defined to be strong, while |ω(a → b, t)| → 0 for *t* → τ indicated a weak effect. In detail, |ω(a → b, t)| → ∞ for *t* → τ indicates that it is |*d/dt* (*f*_*b*_(***M,p***,*t*))| >> |*f*_a_(***M,p***,*t*)|. *Vice versa*, |ω(a → b, *t*)| → 0 for *t* → τ indicates that |*d/dt* (*f*_b_(***M,p***,*t*))| < < |*f*_*a*_(***M,p***,t)|.

### Application example: stress-induced metabolic reprogramming in *Arabidopsis thaliana*

While in the above mentioned example, the calculation and interpretation of ω(t) was demonstrated in context of a previously published kinetic ODE model, another published data set was analyzed by this strategy comprising metabolite levels of the primary and secondary metabolism in *A. thaliana* (Doerfler et al., 2013). In the experiment performed by Doerfler and co-workers, a combined strategy of gas chromatography and liquid chromatography coupled to mass spectrometry was applied in order to reveal a comprehensive picture of metabolic reprogramming during exposure to low temperature and high light intensity. The time period of stress exposure comprised more than 2 weeks which allowed for the analysis of a short- and long-term acclimation response in the metabolome of *A. thaliana*, accession Col-0. A central output of the study was the characterization of metabolomic and regulatory dynamics at the interface of primary and secondary metabolism. The authors observed a fast increase of stress-responsive compounds, e.g., sucrose, which became significant already after 2 days of stress exposure, while the interaction with the secondary metabolism, resulting in biosynthesis of flavonoids, became most significant after 8 days of stress exposure.

To prove the suitability of deriving the absolute value function |ω(*a*→*b, t*)| in order to reveal steps of metabolic regulation within a considered time interval, regression analysis and metabolic functions were calculated for the dataset of Doerfler et al. (2013) and compared to their observations. The metabolic interaction matrix ***Y*** was derived from the metabolic network model which was previously suggested and applied for inverse approximations of the Jacobian matrix (Doerfler et al., 2013). For regression analysis and for integration of metabolic network information we developed and applied a graphical user interface (*FEMTO*, ***F***unctional ***E***valuation of ***M***etabolic ***T***ime series ***O***bservations) which is based on the numerical software environment Matlab®; (http://www.mathworks.com), and which is provided in the supplements together with a user manual (Supplementary Files S1, S2).

To characterize sucrose metabolism, changes of the metabolic function of sucrose were related to changes in sucrose concentrations:

(13) $$\left|\omega \left(Suc\to Suc,t\right)\right|=\left|\frac{\frac{d}{dt}f_{Suc}(M,p,t)}{f_{Suc}(M,p,\;t)}\right|$$

For time-dependent characterization of flavonoid dynamics, changes in the flavonoid (Flav) metabolic function were related to substrate concentration changes, i.e., phenylalanine (Phe) dynamics:

(14) $$\left|\omega \left(Phe\to Flav,t\right)\right|=\left|\frac{\frac{d}{dt}f_{Flav}(M,p,t)}{f_{Phe}(M,p,t)}\right|$$

**Figure 5:**
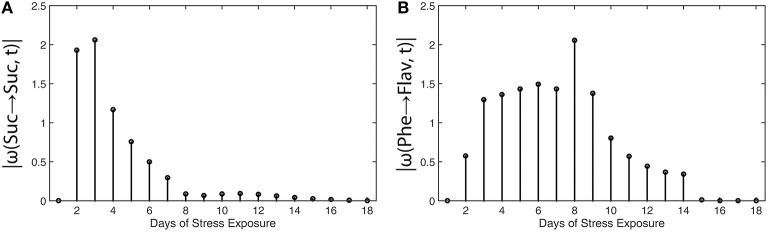
**Absolute value functions of ω(t) for (A) sucrose metabolism and (B) flavonoid biosynthesis in leaves of *Arabidopsis thaliana***. Abscissae indicate days of exposure to low temperature and high-light stress conditions. Detailed information about the calculation is provided in the main text (see Equations 13 and 14). Experimental data were derived from a previous study (Doerfler et al., 2013).

Results of metabolic function analysis and the resulting time course of |ω(*t*)| revealed an early de-regulation of sucrose metabolism during the first 2 days of stress exposure (Figure 5A), while the peak value of |ω(*t*)| for flavonoid biosynthesis occurred delayed after 8 days (Figure 5B).

These findings coincide with the previous findings described by Doerfler and colleagues who applied the method of Granger causality time-series correlation and a covariance-based inverse approximation of Jacobian matrices to reveal strategies of metabolic regulation (Doerfler et al., 2013). Conclusions which have been drawn from the |ω(*t*)| calculation were found to be highly similar to the output of other statistical methods, finally substantiating the validity of the suggested workflow and the derived method to unravel time points of regulatory perturbation in a biochemical system.

## Discussion

Mathematical analysis of biochemical system dynamics represents a central focus of current biomathematical, biochemical and biotechnological research due to the need for methods and algorithms enabling a functional interpretation of experimental data in context of a biochemical network. Particularly, system dynamics which arise due to circadian regulation (Harmer, 2009; Kumar Jha et al., 2015), diurnal metabolic adjustment (Geiger and Servaites, 1994; Pokhilko et al., 2014) or stress-induced metabolic reprogramming (Jozefczuk et al., 2010; Kanshin et al., 2015) are hardly traceable by intuition. Hence, this indicates a strong need for suitable theoretical approaches being capable of resolving and functionally connecting molecular moieties with underlying biochemical regulation.

Various theoretical strategies have addressed this complex issue, providing a comprehensive methodological platform for time-series analysis, dynamic flux balance analysis, kinetic and Boolean modeling (see e.g., Mahadevan et al., 2002; Schelter, 2006; Rohwer, 2012; Steinway et al., 2015). In a recent approach, Willemsen and colleagues have modified the approach of dynamic flux balance analysis by incorporating time-resolved metabolomics measurements (Willemsen et al., 2015). With their extended method, the authors derived an estimate of dynamic flux profiles which allowed them to generate and test hypotheses related to environmentally induced molecular dynamics. In another recent study, a computational approach was suggested to translate metabolomics data into flux information (Cortassa et al., 2015). One main methodological difference between the studies of Willemsen et al. and Cortassa et al. was the extent of kinetic information which was needed to estimate cellular behavior and metabolic fluxes. While Willemsen et al. focused on minimalistic kinetic information, the study of Cortassa and co-workers used a detailed kinetic model of glucose catabolic pathways to derive flux information.

In our presented approach, flux information, which was implicitly derived from spline interpolation, was interpreted only indirectly by comparing time-dependent changes in metabolic functions to concentration changes of biochemical reaction partners. This procedure revealed information about a rate which was interpreted in terms of metabolic functions related to concentration changes in a substrate or co-substrate. Comparing derived results to other methods, it was shown that changes in ratios of second- to first-order derivatives between functionally connected variables potentially reveal time points of regulatory perturbation within a biochemical interaction. Hence, these observed perturbations might indicate a change in enzymatic activity, protein abundance, or allosteric regulation ultimately leading to a change in the metabolic functions.

The information content of the introduced time-dependent functions ω(*t*) is related to entries of the Jacobian matrix ***J*** (see Equation 3) indicating the dynamics of metabolic functions with respect to (small) concentration changes at a certain steady state. This theoretical connection of ***J*** and ω(*t*) at a considered time point t_0_ might be illustrated in a simple first-order reaction scheme.

(15) $$A\overset{k}{\to }B$$

Here, substance *A* is interconverted into substance *B*, and the reaction velocity is characterized by the rate constant *k*. The time-dependent change in concentration of *A* equals *dA/dt* = −*k*·*A*. Hence, a general solution of this ODE is given by *A(t)* = *A*_0_*e*^−kt^ which finally yields *J*_11_(*t*_0_) = ω(*A*→*A, t*_0_) = −k.

With this, the information of ω(*t*) becomes comparable to entries of the Jacobian matrix ***J***. Yet, in contrast to entries of ***J***, characterizing dynamic properties of a metabolic steady state (*d/dt* ***M***(*t*) = 0), functions ω(*t*) were derived from a time series of experimental data and might rather be valid for a non-infinitesimal than for an infinitesimal time frame. While for lim_*t*→*t*_0__|ω(*t*)|, |ω(*t*)| might be assumed to approach entries of ***J***, this was not tested in the present study and would need experimental validation. In addition, while a connection, and probable correlation, to other molecular levels, such as the proteome or transcriptome, was not experimentally analyzed, this might be a promising target for analysis in future studies. However, the incorporation of an interaction matrix, which, in the present study, was derived from a previously published reaction network, and which might be derived from genome-scale metabolic reconstruction works in future studies (Weckwerth, 2011; King et al., 2015), provides direct evidence for the biochemical and physiological relevance of the performed theoretical analysis.

While our results indicate a realistic and biochemically interpretable output of the presented method, limitations of application might occur due to several reasons. First, the presented method significantly depends on the knowledge about the biochemical network structure and involved regulatory interactions, e.g., feedback inhibition or feedforward activation. Although regression analysis of time series data might be performed for all network components independently, deriving a reliable biochemical interaction matrix ***Y*** is essential to reveal realistic information about time-dependent changes in metabolic interactions. A second central prerequisite for a meaningful regression analysis is the design of an adequate experimental setup. This comprises the number of biological (independent) replicates as well as the number and interval of sampling points. It is hardly possible to generalize a number of replicates or sampling points due to heterogeneous technical or environmental fluctuations which are introduced by different analytical techniques, growth conditions or sample types. Yet, spanning various experimental scenarios, it might be generalized that the interval of sampling points is crucial to be able to discriminate between metabolic fast or short-term responses and slow or long-term responses. Particularly to resolve fast metabolic regulation, a narrow sampling interval is needed in order to prevent any over-interpretation of regression analysis and related derivatives. Comparing the presented approach to methods of metabolic modeling, a third major limitation is the missing predictive output by model simulations. For example, enzyme kinetic models of metabolism aim at going beyond the time interval of measured rate constants or metabolite concentrations to predict changes in system dynamics under changing environmental conditions or due to a mutated gene. However, although our presented method cannot afford this simulation output, time-dependent changes within the considered time interval might indicate regulatory bottlenecks and kinetic information supporting the numerical solution and simulation of metabolic ODE models.

In summary, the suggested approach intends to promote the functional interpretability of metabolic time series data in context of metabolic network information. Particularly with regard to multidimensional metabolomics data sets, this might unravel strategies of complex biochemical regulation and might overcome some limitations in the generation of testable hypotheses as we have discussed previously (Nägele and Weckwerth, 2012). Finally, the direct integration of biochemical network information with experimental data promises to enable the functional interpretation and the causal connection of various levels of molecular organization.

## Materials and methods

The described procedure of data analysis, spline interpolation and graphical representation was performed within the numerical software environment Matlab®;. A Matlab-based graphical user interface (FEMTO, *F*unctional *E*valuation of *M*etabolic *T*ime series *O*bservations) was developed and is provided, together with a user manual, in the supplements (Supplementary Files S1, S2).

## Author contributions

TN, LF, MN, and JW performed data analysis, statistics, wrote the source code of the graphical user interface and wrote the paper. TN and WW conceived the study and wrote the paper. All authors read and approved the final version of the manuscript.

## Funding

This work was supported by the Austrian Science Fund, FWF, Project P 26342 and Project I 2071.

### Conflict of interest statement

The authors declare that the research was conducted in the absence of any commercial or financial relationships that could be construed as a potential conflict of interest.

## Supplementary material

Supplementary File S1 **Matlab code-file and test data file**.

Supplementary File S2 **Manual for the graphical user interface FEMTO 1.0**.
