# Supplementary material for: A Strategy for Functional Interpretation of Metabolomic Time Series Data in Context of Metabolic Network Information
Source: Front Mol Biosci. 2016 Mar 7;3:6. doi: 10.3389/fmolb.2016.00006 (PMC4779852; doi:10.3389/fmolb.2016.00006)
Supplement: Supplementary File S1 — Matlab code-file and test data file. [file DataSheet1.ZIP › Supplement S1 FEMTO_package/Supplement S1 FEMTO_package/FEMTO/FEMTO_Manual.pdf]

Manual

FEMTO 1.0

–

Functional Evaluation of Metabolic Time series Observations

Developed by:

Thomas Nägele, Lisa Fürtauer, Matthias Nagler, Jakob Weiszmann and Wolfram Weckwerth

Contact Information:

University of Vienna
Department of Ecogenomics and Systems Biology
Faculty of Life Sciences
Althanstr. 14
1090 Vienna, Austria

Email: Thomas.Naegele@univie.ac.at

URL: http://www.univie.ac.at/mosys/

Table of Contents

I. FEMTO 1.0: a Matlab®[1]-based graphical user interface 2

II. Requirements 3

III. Execution of FEMTO 1.0 3

IV. Application of FEMTO 1.0 4

Data Import: Panel I 4

Regression Analysis: Panel II 4

Differentiation: Panel III 6

Interaction Analysis: Panel IV 6

Interaction Parameters: Panel V 7

The File Panel 8

Example Data 8

I. FEMTO 1.0: a Matlab®[1]-based graphical user interface

A functional connection and interpretation of metabolic time series data in context of metabolic networks represents one of the central research topics on systems biology. The motivation of the development of FEMTO (**Fig. 1**) was to provide an intuitive graphical user interface enabling the efficient integration of experimental time series data and metabolic network information.

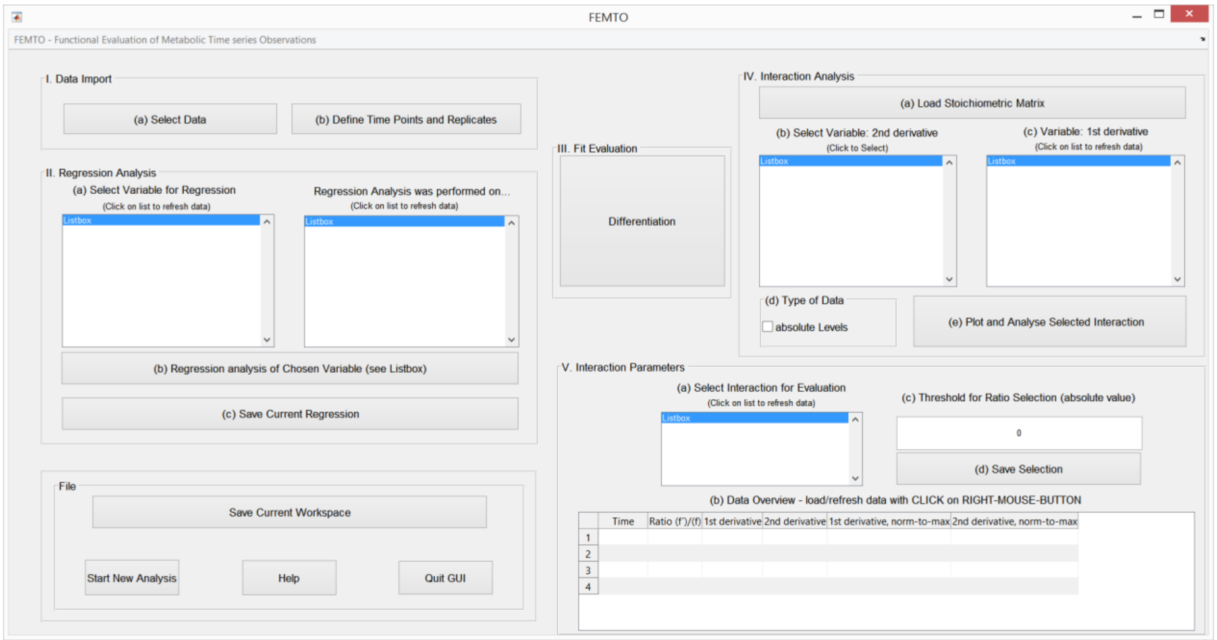

Figure 1: The graphical user interface FEMTO 1.0

This manual is intended to guide the user through the evaluation process of time series data. The main steps which have to be performed during data import, regression analysis and evaluation are graphically comprised by panels and indicated by a sequence of Roman numerals (**Steps I. – V.**; **Fig. 1**). During each of those main steps, several minor steps have to be performed which are indicated by lowercase letters (see **Fig. 1**). To assist the user, buttons will change their colour from grey to cyan as soon as they are tapped.

We hope that the following chapters are helpful in context of data evaluation and toolbox application. However, in case of open questions, problems with version compatibility or suggestions for improvement, please do not hesitate to contact us using the contact information provided on the title page of this document.

The FEMTO developer-Team

[1] www.mathworks.com

II. Requirements

FEMTO 1.0 is a MATLAB®-based graphical user interface and was developed on MATLAB® R2014b (8.4.0) 64-bit(win64). The MathWorks® Curve Fitting Toolbox™ is needed for the regression analysis which is part of the FEMTO workflow.

III. Execution of FEMTO 1.0

1. Save and unpack the FEMTO package to a location on your hard drive
2. Open folder \...**\FEMTO** in the *Current Folder* of your MATLAB® window.
3. Type 'FEMTO' in the *Command Window* of MATLAB®. Alternatively, you can start FEMTO 1.0 by choosing 'FEMTO.m' with the left mouse-button and tapping F9 on your keyboard.
4. Remark: to save the path of the folder 'FEMTO' to your MATLAB® search path, use the MATLAB® *pathtool*.

IV. Application of FEMTO 1.0

Data Import: Panel I

Clicking on the button **'(a) Select Data'** allows the selection of a file containing time series data. As soon as the data file has been chosen, an import wizard opens which enables the user to specify the data import.

IMPORTANT: Data have to be organized row-wise (rows -> variables; columns -> replicates for each time point). Please have a look at the provided example data set for correct data organization (\FEMTO\misc\example_data.xlsx). If the data are not organized correctly, the evaluation will fail!

In the next step **'(b) Define Time Points and Replicates'** the user is asked to provide the time points and number of replicates at each of these time points. Time points have to be entered in the following format: **[TP1 TP2 TP3 …]**. (see **Fig. 5**).

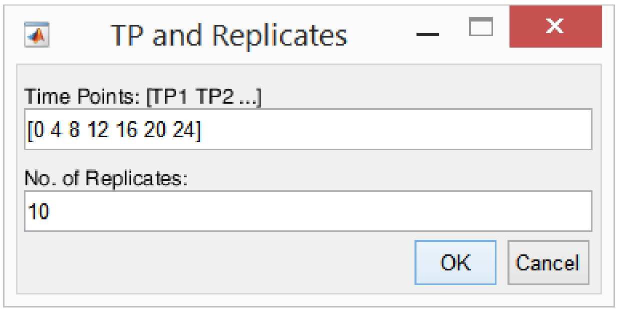

Figure 2: Prompt for definition of time points and number of replicates. In this example, the time points are 0,4,8,12,16,20,24 (arbitrary time units) and the number of replicates is 10.

Regression Analysis: Panel II

To select a variable for regression, click on listbox **'(a) Select Variable for Regression'**. All variables which have been imported should become visible in the listbox. Select one variable from the list and click on **'(b) Regression analysis of Chosen Variable (see Listbox)'**.

The window of the MathWorks® Curve Fitting Toolbox™ opens. Choose '**timepoints**' for X data, the selected variable for Y data and '**Selected_Weights**' for Weights. The regression method can be chosen by the user. We recommend the '**Smoothing Spline**' with default settings(**Fig. 6**).

Save the fitting results by clicking on '**Fit -> Save to Workspace…**'. Please use the default settings for saving the fitting results (**Fig. 7**)! Having saved the results to the workspace, go back to the FEMTO main window and click on **'(c) Save Current Regression**' – this makes the results applicable in FEMTO.

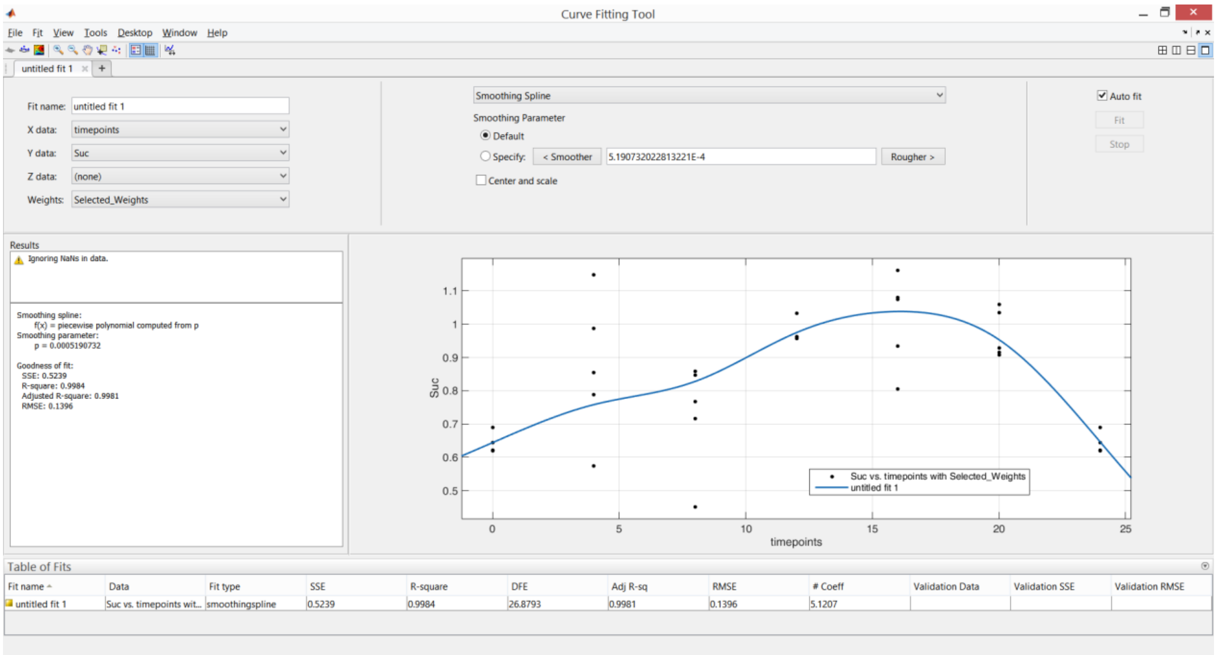

Figure 3: Settings for the regression analysis in the MathWorks® Curve Fitting Toolbox™.

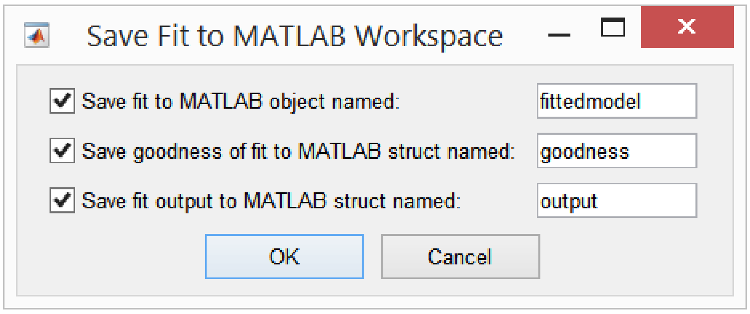

Figure 4: Default settings for saving the regression results.

The above described procedure has to be repeated for all variables. To provide a better overview of successfully fitted and saved variables, the user can click on the listbox '**Regression Analysis was performed on…**'. All analysed and saved variables are displayed here.

IMPORTANT: For starting a new regression analysis, it is necessary that the user performs all steps again. Entries in the Curve Fitting Toolbox™ **are not automatically updated**, i.e. to load the correct weights for the regression analysis, the user has to choose '**Selected Weights**' for each individual regression (**Fig. 8**)!

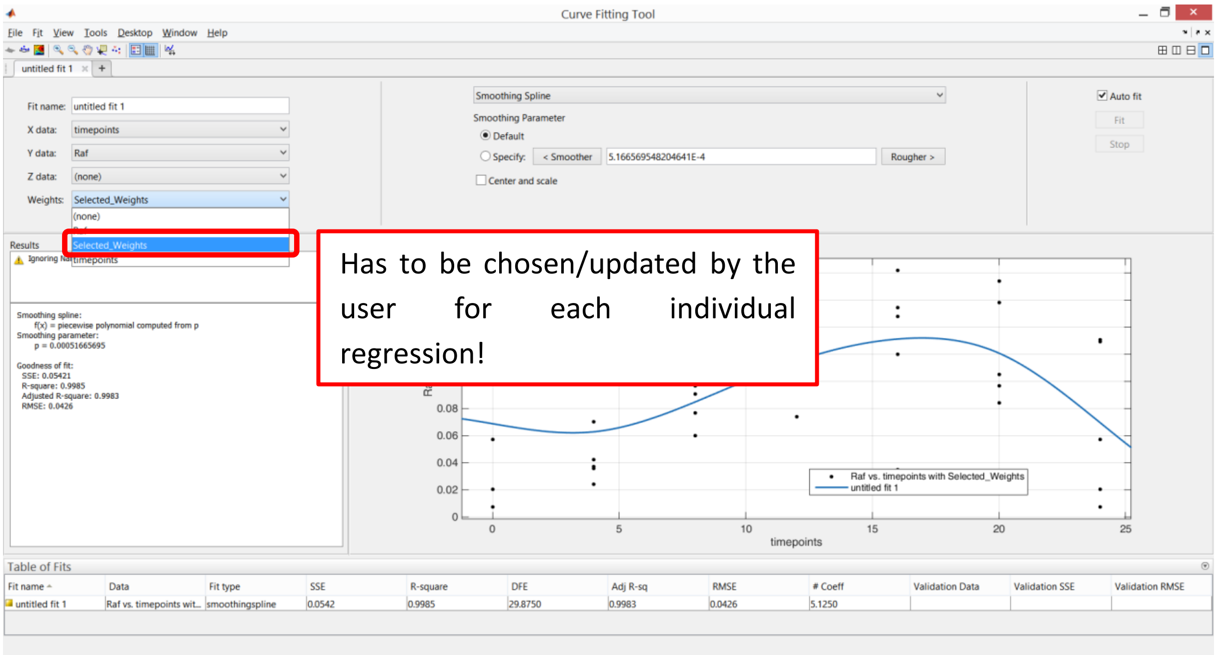

Figure 5: It is important to choose the 'Selected_Weights' for each individual regression separately. The Curve Fitting Toolbox™ will not automatically update the weight information!

Differentiation: Panel III

Clicking on this button performs all data evaluation steps which are necessary for the following interaction analysis.

Interaction Analysis: Panel IV

The first step of the interaction analysis is to load the stoichiometric matrix of the metabolic network by clicking on **'(a) Load Stoichiometric Matrix'**. An import wizard window opens which allows the user to choose the stoichiometric matrix information. Following the loading process, the user can select a combination of variables to be analysed by their 1st and 2nd derivative (**'(b) Select Variable: 2nd derivative'** and **'(c) Select Variable: 1st derivative'**). All combinations which can be chosen from both listboxes are derived from the network information.

IMPORTANT: Variable names, i.e. metabolites, in the stoichiometric matrix have to be named according to the names in the regression analysis! **Variable names are compared in a case-sensitive manner**, i.e. lower-case and capital letters are distinguished! If variables are not named equivalently, interaction analysis is not possible!

Comparison of the metabolic functions and their derivatives can be plotted by clicking on button **‘(e) Plot and Analyse Selected Interaction’**. If absolute levels of variables are available, the user can indicate this in check-box **‘(d) Type of Data’**. In case of absolute levels, absolute values of derivatives are used for ratio calculation. In case of relative levels, the ratio is calculated using derivatives normalized to the (absolute) maximum value. The plot is provided as a MATLAB® figure file and can be saved or modified.

Interaction Parameters: Panel V

In this panel, the plotted ratio **(f’)/(f)** (which equals the ratio **Variable 2nd derivative/Variable 1st derivative**), and the fit evaluation data is provided in a table **‘(b) Data Overview – load/refresh data with CLICK on RIGHT-MOUSE-BUTTON’**.

The metabolic interaction can be selected via the listbox **‘(a) Select Interaction for Evaluation’**. The time course of the chosen variable is then evaluated with respect to the selection in panel IV, listbox **‘(b) Select Variable: 2nd derivative’** (**Fig. 9**).

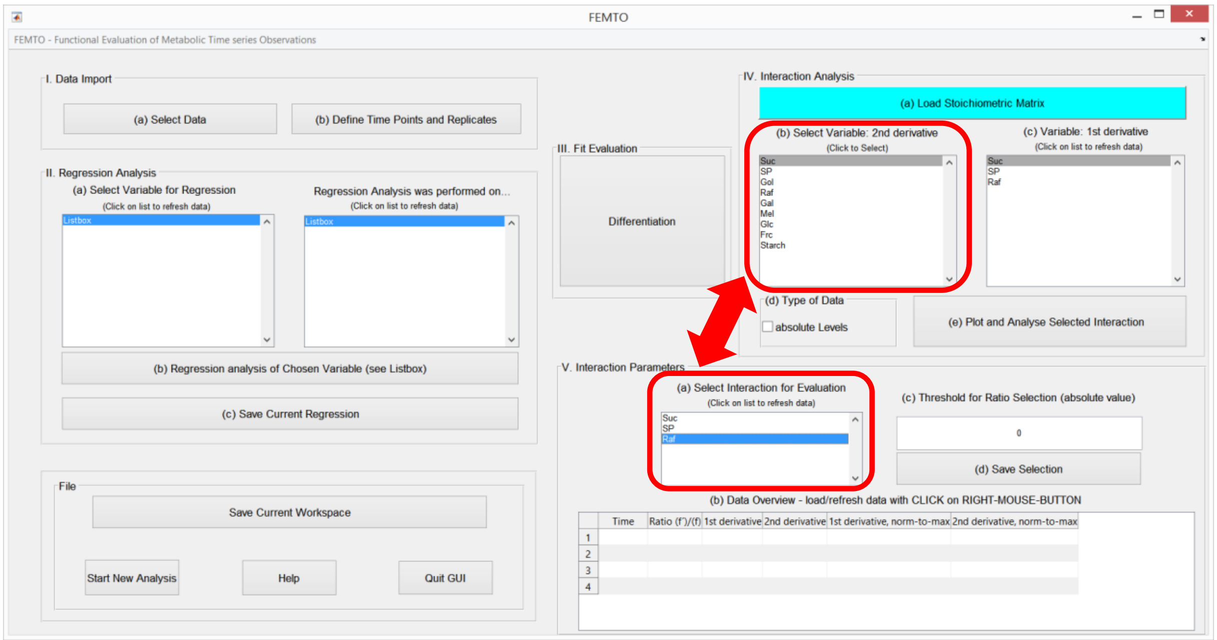

Figure 6: Example for selecting a metabolic interaction for evaluation. In this example, the 2nd derivative of the regression for the variable ‘Suc’ is compared to the first derivative of the regression for variable ‘Raf’.

To derive the numerical values of ratios and derivative, click on table ‘(b)’ with the right mouse button. Rows are sorted in an ascendant manner of entries in column 2 (**Ratio (f’)/(f)**). By clicking on button ‘**(d) Save Selection**’ the table can be saved to a MATLAB® workspace file (*.mat), to an xlsx-file and/or to a csv-file. As a default setting, the threshold

for ratio selection is set to 0, i.e. the complete table is saved to a file. Beyond, the user is able to save only those table entries which have a higher (absolute) ratio than the given threshold. The threshold (Format: decimal point) can be modified by typing a certain value in textbox **'(c) Threshold for Ratio Selection (absolute value)'**.

The File Panel

At each point of of data evaluation, results can be saved to a MATLAB® workspace using the button **'Save Current Workspace'**. The workspace can be used for further data analysis in MATLAB®. A restart of FEMTO 1.0 is possible by clicking on the button 'Start New Analysis'.

IMPORTANT: a restart irreversibly deletes all information and evaluation results from the current analysis!

Example Data

A data set is provided to test the workflow of FEMTO 1.0 ('Example_Data.xlsx'). The data set contains experimental data (3 replicates, 6 time points, 9 variables) and a stoichiometric matrix which can be loaded and applied.
